# Supplementary material for: Energetic instability of passive states in thermodynamics
Source: Nat Commun. 2017 Dec 1;8:1895. doi: 10.1038/s41467-017-01505-4 (PMC5711874; doi:10.1038/s41467-017-01505-4)
Supplement: Supplementary file 1 — Supplementary Information [file 41467_2017_1505_MOESM1_ESM.pdf]

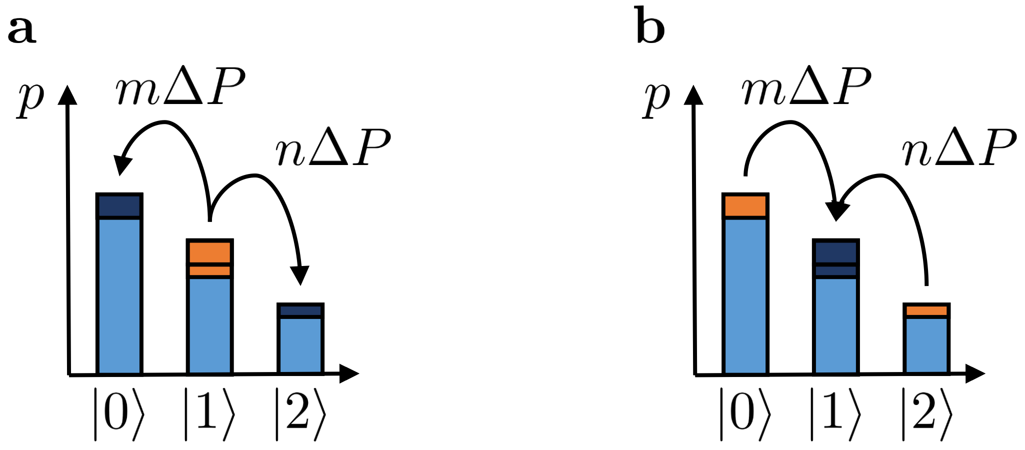

Supplementary Figure 1. The action of the cycle $S_{m,n}$ on a qutrit passive state. (**a**) The action of the cycle $S_{m,n}$ on the spectrum of a passive state, when work is extracted and the Hamiltonian $H_{\rm P}$ is such that $m\,\Delta E_{10} > n\,\Delta E_{21}$. The probability of occupation of $|1\rangle_{\rm P}$ is reduced by $(m+n)\,\Delta P$ (orange), where $\Delta P > 0$, while the probabilities of occupation of $|0\rangle$ and $|1\rangle$ increase, respectively, by $m\,\Delta P$ and $n\,\Delta P$ (dark blue). (**b**) The action of the same cycle on the spectrum of a passive state, when work is extracted and the Hamiltonian $H_{\rm P}$ is such that $m\,\Delta E_{10} < n\,\Delta E_{21}$. The map acts on the system in the opposite way compared to the previous scenario.

Supplementary Note 1. GENERAL CYCLE FOR WORK EXTRACTION FROM PASSIVE STATES

We present in full details the general cycle needed to extract work from a qutrit system described by a passive state. Work extraction is achieved through the interaction between a qudit ancilla (the thermal machine) and the main qutrit system. This qutrit system has Hamiltonian

$$H_{\rm P} = E_0 \left|0\right\rangle\left\langle 0\right|_{\rm P} + E_1 \left|1\right\rangle\left\langle 1\right|_{\rm P} + E_2 \left|2\right\rangle\left\langle 2\right|_{\rm P}\,, \tag{1}$$

and we define the energy gap as $\Delta E_{10} = E_1 - E_0 > 0$ and $\Delta E_{21} = E_2 - E_1 > 0$. The state of the system is passive, meaning that no energy can be extracted with unitary operations, and we can write it as a classical state

$$\rho_{\rm P} = p_0 \left|0\right\rangle\left\langle 0\right|_{\rm P} + p_1 \left|1\right\rangle\left\langle 1\right|_{\rm P} + p_2 \left|2\right\rangle\left\langle 2\right|_{\rm P}\,, \tag{2}$$

where $p_0 \geq p_1 \geq p_2$ (which is a direct consequence of the no-energy-extraction condition).

The machine we introduce is a d-level system with a trivial Hamiltonian, described by the state

$$\rho_{\rm M} = \sum_{j=0}^{d-1} q_j \left|j\right\rangle\left\langle j\right|_{\rm M}\,. \tag{3}$$

We operate over system and machine with a unitary operation composed by multiple swaps. In particular, we first perform $m-1$ swaps between the pair of states $(|0\rangle_{\rm P}\,, |1\rangle_{\rm P})$ and the pairs $\{(|j\rangle_{\rm M}\,, |j+1\rangle_{\rm M})\}_{j=0}^{m-2}$, followed by a swap between the same pair of states of the system and the pair $(|m-1\rangle_{\rm M}\,, |m+n-1\rangle_{\rm M})$ of the machine. Then, we perform $n-1$ swaps between the pair $(|1\rangle_{\rm P}\,, |2\rangle_{\rm P})$ and the pairs $\{(|j\rangle_{\rm M}\,, |j+1\rangle_{\rm M})\}_{j=m}^{m+n-2}$, followed by a swap between the same system's states and the pair $(|0\rangle_{\rm M}\,, |m\rangle_{\rm M})$. In order to perform this cycle, the dimension of the catalyst has to be at least equal to $m+n$, and indeed in the following we fix $d = m+n$. The unitary we want to apply is

$$S_{m,n} = S_{(1,2)}^{(0,m)} \circ S_{(1,2)}^{(m,m+1)} \circ S_{(1,2)}^{(m+1,m+2)} \circ \ldots \circ S_{(1,2)}^{(m+n-2,m+n-1)} \circ S_{(0,1)}^{(m-1,m+n-1)} \circ S_{(0,1)}^{(m-2,m-1)} \circ S_{(0,1)}^{(m-3,m-2)} \circ \ldots \circ S_{(0,1)}^{(0,1)}\,, \tag{4}$$

where the operation $S_{(a,b)}^{(c,d)}$ is a swap between system and machine, performing the permutation $|a\rangle_{\rm P}\,|d\rangle_{\rm M} \leftrightarrow |b\rangle_{\rm P}\,|c\rangle_{\rm M}$.

For the given unitary evolution we can easily evaluate the final state of the global system. This final state presents classical correlations between system and machine, but in the following we only consider the marginal states for system and machine, which are the sole information we need. In fact, the energy of the global system solely depends on the Hamiltonian $H_{\rm P}$ of the system (and therefore only on the local state of the system), as the machine has a trivial

Hamiltonian, and we do not have an interaction term $H_{\rm int}$. Moreover, in order for the machine to be re-usable on a new system, we only need its local initial and final states to be equal, and the correlations with the old systems do not affect the engine. The final state of the system is

$$\begin{aligned}
\tilde{\rho}_{\rm P} &= {\rm Tr}_{\rm M}\left[S_{m,n}\left(\rho_{\rm P}\otimes\rho_{\rm M}\right)S^{\dagger}_{m,n}\right] \\
&= \left(p_0+\sum_{j=1}^{m-1}\left(p_1q_{j-1}-p_0q_j\right)+\left(p_1q_{m-1}-p_0q_{m+n-1}\right)\right)\left|0\right\rangle\left\langle 0\right|_{\rm P} \\
&+\left(p_1-\sum_{j=1}^{m-1}\left(p_1q_{j-1}-p_0q_j\right)-\left(p_1q_{m-1}-p_0q_{m+n-1}\right)-\sum_{j=m+1}^{m+n-1}\left(p_1q_j-p_2q_{j-1}\right)-\left(p_1q_m-p_2q_0\right)\right)\left|1\right\rangle\left\langle 1\right|_{\rm P} \\
&+\left(p_2+\sum_{j=m+1}^{m+n-1}\left(p_1q_j-p_2q_{j-1}\right)+\left(p_1q_m-p_2q_0\right)\right)\left|2\right\rangle\left\langle 2\right|_{\rm P}\,, \qquad (5)
\end{aligned}$$

while the final state of the machine is

$$\begin{aligned}
\tilde{\rho}_{\rm M} &= {\rm Tr}_{\rm P}\left[S_{m,n}\left(\rho_{\rm P}\otimes\rho_{\rm M}\right)S^{\dagger}_{m,n}\right] \\
&= \left(p_0q_0+p_0q_1+p_1q_m\right)\left|0\right\rangle\left\langle 0\right|_{\rm M}+\sum_{j=1}^{m-2}\left(p_1q_{j-1}+p_0q_{j+1}+p_2q_j\right)\left|j\right\rangle\left\langle j\right|_{\rm M} \\
&+\left(p_1q_{m-2}+p_0q_{m+n-1}+p_2q_{m-1}\right)\left|m-1\right\rangle\left\langle m-1\right|_{\rm M}+\left(p_0q_m+p_2q_0+p_1q_{m+1}\right)\left|m\right\rangle\left\langle m\right|_{\rm M} \\
&+\sum_{j=m+1}^{m+n-2}\left(p_0q_j+p_2q_{j-1}+p_1q_{j+1}\right)\left|j\right\rangle\left\langle j\right|_{\rm M}+\left(p_1q_{m-1}+p_2q_{m+n-2}+p_2q_{m+n-1}\right)\left|m+n-1\right\rangle\left\langle m+n-1\right|_{\rm M}\,. \qquad (6)
\end{aligned}$$

As we stated above, in order for the machine to be re-usable we need its final local state $\tilde{\rho}_{\rm M}$ to be equal to the initial one $\rho_{\rm M}$. Correlations with the system do not invalidate the re-usability, as we always discard the system after the cycle, and we take a new copy to repeat the process. In this way, we can extract work from a reservoir of passive states by acting on them individually. The constraint of an equal initial and final state of the machine provides the following set of equalities,

$$q_0 = p_0q_0+p_0q_1+p_1q_m \qquad (7)$$

$$q_j = p_1q_{j-1}+p_0q_{j+1}+p_2q_j \quad ; \quad j=1,\ldots,m-2 \qquad (8)$$

$$q_{m-1} = p_1q_{m-2}+p_0q_{m+n-1}+p_2q_{m-1} \qquad (9)$$

$$q_m = p_0q_m+p_2q_0+p_1q_{m+1} \qquad (10)$$

$$q_j = p_0q_j+p_2q_{j-1}+p_1q_{j+1} \quad ; \quad j=m+1,\ldots,m+n-2 \qquad (11)$$

$$q_{m+n-1} = p_1q_{m-1}+p_2q_{m+n-2}+p_2q_{m+n-1}, \qquad (12)$$

which, if solved, allow for the probability distribution of the state of the machine to be expressed in terms of the passive state $\rho_{\rm P}$.

Work extracted and activable passive states

In our framework, we do not explicitly account for a battery, that is, an additional system with a specific Hamiltonian, able to account for any energy exchange between system and machine. Instead, we implicitly assume the battery to be present, so that any change in the average energy of the system is thought as some energy flowing from (or to) the battery. In particular, if the average energy of the system decreases, then the battery is storing this energy, while when the average energy of the system increases, the battery is providing it. All the energy coming from (or going to) the battery is accounted as work. Under this assumptions, the amount of work we extract during one cycle is given by the changing in the average energy of the system, that is

$$\Delta W = {\rm Tr}_{\rm P}\left[H_{\rm P}\left(\rho_{\rm P}-\tilde{\rho}_{\rm P}\right)\right], \qquad (13)$$

where $\rho_{\rm P}$ is the initial passive state, and $\tilde{\rho}_{\rm P}$ is the final state, whose probability distribution is $\{p'_0,p'_1,p'_2\}$. We can express the amount of extracted work in terms of the energy gaps of the Hamiltonian $H_{\rm P}$, as

$$\Delta W = \Delta E_{10}\left(p'_0-p_0\right)-\Delta E_{21}\left(p'_2-p_2\right), \qquad (14)$$

where this expression has been obtained by applying the normalisation constraint to the initial and final state of the system.

If we replace the probability distribution of the final state of the system, Supplementary Eq. (5), into the expression of extracted work, Supplementary Eq. (14), we obtain that

$$\Delta W = \Delta E_{10} \left(\sum_{j=1}^{m-1} (p_1 q_{j-1} - p_0 q_j) + (p_1 q_{m-1} - p_0 q_{m+n-1}) \right) - \Delta E_{21} \left(\sum_{j=m+1}^{m+n-1} (p_1 q_j - p_2 q_{j-1}) + (p_1 q_m - p_2 q_0) \right). \tag{15}$$

This expression can be highly simplified if we use the properties of the probability distribution of the machine, Supplementary Eqs. (7) to (12). In particular, from Supplementary Eq. (8) we find that

$$p_1 q_{j-1} - p_0 q_j = p_1 q_j - p_0 q_{j+1} \quad ; \quad \forall\, j = 1, \ldots, m-2, \tag{16}$$

while from (9) we have that

$$p_1 q_{m-2} - p_0 q_{m-1} = p_1 q_{m-1} - p_0 q_{m+n-1}. \tag{17}$$

Together, these equations reduce the first bracket of Supplementary Eq. (15) into a single term,

$$\sum_{j=1}^{m-1} (p_1 q_{j-1} - p_0 q_j) + (p_1 q_{m-1} - p_0 q_{m+n-1}) = m\,(p_1 q_{m-1} - p_0 q_{m+n-1}). \tag{18}$$

If we consider Supplementary Eq. (11), instead, we find that

$$p_1 q_j - p_2 q_{j-1} = p_1 q_{j+1} - p_2 q_j \quad ; \quad \forall\, j = m+1, \ldots, m+n-2, \tag{19}$$

while Supplementary Eq. (10) implies that

$$p_1 q_{m+1} - p_2 q_m = p_1 q_m - p_2 q_0. \tag{20}$$

These two equations simplify the second bracket of Supplementary Eq. (15),

$$\sum_{j=m+1}^{m+n-1} (p_1 q_j - p_2 q_{j-1}) + (p_1 q_m - p_2 q_0) = n\,(p_1 q_{m+n-1} - p_2 q_{m+n-2}). \tag{21}$$

We can now use Supplementary Eq. (12) to show that

$$p_1 q_{m-1} - p_0 q_{m+n-1} = p_1 q_{m+n-1} - p_2 q_{m+n-2}, \tag{22}$$

which allows us to express the work we extract as

$$\Delta W = (m\,\Delta E_{10} - n\,\Delta E_{21})\,(p_1 q_{m+n-1} - p_2 q_{m+n-2}). \tag{23}$$

From the above equation we notice that the work extracted is factorised into an Hamiltonian contribution and another contribution associated with the probability distribution of the passive state. Then, for a given Hamiltonian H_{P} such that $m\,\Delta E_{10} > n\,\Delta E_{21}$, we will find that certain passive states allow for work extraction (the ones in which $p_1 q_{m+n-1} > p_2 q_{m+n-2}$), while others do not. Therefore, for every given Hamiltonian (that is, every ΔE_{10} and ΔE_{21}) and for every given cycle (that is, every n and m), we find that the set of passive states is divided into two subsets, the ones which allow for work extraction (we can call them activable states), and the ones which do not. In the following we will express the probability distribution of ρ_{M} in terms of the probability distribution of the passive state, so as to define these two subsets for each Hamiltonian and cycle.

As a first step, we want to express the first $m-2$ elements of the sequence $\{q_j\}_{j=0}^{m-1}$ in terms of last two elements, q_{m-2} and q_{m-1}. Moreover, we express the first $n-2$ elements of $\{q_j\}_{j=m}^{m+n-1}$ in terms of q_{m+n-2} and q_{m+n-1}. This can be done by utilising the equalities given in Supplementary Eqs. (8) and (11), which we recast in the following way.

$$q_j = \left(1 + \frac{p_0}{p_1}\right) q_{j+1} - \frac{p_0}{p_1}\, q_{j+2} \quad ; \quad \forall\, j = 0, \ldots, m-3, \tag{24}$$

$$q_j = \left(1 + \frac{p_1}{p_2}\right) q_{j+1} - \frac{p_1}{p_2}\, q_{j+2} \quad ; \quad \forall\, j = m, \ldots, m+n-3. \tag{25}$$

It can be proved (see Supplementary Note 6) that the elements of the sequences can be expressed as

$$q_j = \mathrm{T}_1\left(m-(j+2)\right) q_{m-2} - \frac{p_0}{p_1}\,\mathrm{T}_1\left(m-(j+3)\right) q_{m-1} \quad ; \quad \forall\, j = 0,\ldots,m-3, \tag{26}$$

$$q_j = \mathrm{T}_2\left(m+n-(j+2)\right) q_{m+n-2} - \frac{p_1}{p_2}\,\mathrm{T}_2\left(m+n-(j+3)\right) q_{m+n-1} \quad ; \quad \forall\, j = m,\ldots,m+n-3, \tag{27}$$

where $\mathrm{T}_1(h) = \sum_{l=0}^{h}\left(\frac{p_0}{p_1}\right)^l$ and $\mathrm{T}_2(h) = \sum_{l=0}^{h}\left(\frac{p_1}{p_2}\right)^l$.

We can now express, using Supplementary Eqs. (9) and (12), the elements q_{m-2} and q_{m-1} in terms of q_{m+n-2} and q_{m+n-2}. From Supplementary Eq. (9) we obtain that

$$q_{m-2} = \mathrm{T}_1(2)\, q_{m+n-1} - \frac{p_2}{p_1}\,\mathrm{T}_1(1)\, q_{m+n-2}. \tag{28}$$

From Supplementary Eq. (12), instead, we get that

$$q_{m-1} = \mathrm{T}_1(1)\, q_{m+n-1} - \frac{p_2}{p_1}\,\mathrm{T}_1(0)\, q_{m+n-2}. \tag{29}$$

Then, we can finally express q_{m+n-2} in terms of q_{m+n-1} through Supplementary Eq. (10), and we obtain

$$q_{m+n-2} = D(m,n)\, q_{m+n-1}, \tag{30}$$

where the coefficient $D(m,n)$ is defined as

$$D(m,n) = \frac{p_1}{p_2}\,\frac{\mathrm{T}_1(m) + \frac{p_1}{p_2}\mathrm{T}_2(n-2)}{\mathrm{T}_1(m-1) + \frac{p_1}{p_2}\mathrm{T}_2(n-1)}. \tag{31}$$

Thanks to the above result, we can express the overall probability distribution of ρ_{M} in terms of the occupation probability of the state $|m+n-1\rangle_{\mathrm{M}}$. Thus, we have that

$$q_j = \left(\mathrm{T}_1(m-j) - \frac{p_2}{p_1}\,D(m,n)\,\mathrm{T}_1\left(m-(j+1)\right)\right) q_{m+n-1} \quad ; \quad j = 0,\ldots,m-1, \tag{32}$$

$$q_j = \left(\mathrm{T}_2\left(m+n-(j+2)\right)\, D(m,n) - \frac{p_1}{p_2}\,\mathrm{T}_2\left(m+n-(j+3)\right)\right) q_{m+n-1} \quad ; \quad j = m,\ldots,m+n-3, \tag{33}$$

$$q_{m+n-2} = D(m,n)\, q_{m+n-1}, \tag{34}$$

where it is possible to show that each q_j, with $j = 0,\ldots,m+n-2$, is positive if q_{m+n-1} is positive (see the technical result 3). From the normalisation condition it then follows that the sequence $\{q_j\}_{j=0}^{m+n-1}$ is a proper probability distribution. Moreover, the normalisation condition allows us to evaluate q_{m+n-1} as a function of the probability distribution of the passive state ρ_{P},

$$q_{m+n-1} = \frac{\mathrm{T}_1(m-1) + \frac{p_1}{p_2}\mathrm{T}_2(n-1)}{\left(\mathrm{T}_1(m) + \frac{p_1}{p_2}\mathrm{T}_2(n-2)\right)^2 + \left(\left(\frac{p_1}{p_2}\right)^n - \left(\frac{p_0}{p_1}\right)^m\right)\left(\sum_{j=0}^{m}\mathrm{T}_1(j) - \frac{p_1}{p_2}\sum_{j=0}^{n-3}\mathrm{T}_2(j)\right)}. \tag{35}$$

From Supplementary Eq. (35) we can express all the other elements of $\{q_j\}_{j=0}^{m+n-1}$ in terms of the probability distribution of ρ_{P}.

We can now further characterise the amount of work extracted during our cycle. In fact, if we apply Supplementary Eq. (34) into Supplementary Eq. (23), we obtain

$$\Delta W = \left(m\,\Delta E_{10} - n\,\Delta E_{21}\right)\frac{p_1\left(\left(\frac{p_1}{p_2}\right)^n - \left(\frac{p_0}{p_1}\right)^m\right)}{\mathrm{T}_1(m-1) + \frac{p_1}{p_2}\mathrm{T}_2(n-1)}\, q_{m+n-1}, \tag{36}$$

where the sign of ΔW depends on the sole terms $(m\,\Delta E_{10} - n\,\Delta E_{21})$ and $\left(\left(\frac{p_1}{p_2}\right)^n - \left(\frac{p_0}{p_1}\right)^m\right)$, since the other factors are always positive. Thus, for each cycle, we can characterise which passive states can be activated by that cycle, that is, which states allow for work extraction during the cycle. The subset of activable states is

$$R^+_{m,n} = \left\{\rho_{\mathrm{P}} \text{ passive} \;\middle|\; \left(\frac{p_1}{p_2}\right)^n > \left(\frac{p_0}{p_1}\right)^m \text{ when } m\,\Delta E_{10} - n\,\Delta E_{21} > 0 \right.$$
$$\left. \vee \left(\frac{p_1}{p_2}\right)^n < \left(\frac{p_0}{p_1}\right)^m \text{ when } m\,\Delta E_{10} - n\,\Delta E_{21} < 0\right\}, \tag{37}$$

where this region clearly depends on the Hamiltonian of the system H_{P}, and on the number of swaps performed during the cycle, m and n.

The final state of the system

Let us consider the final state of the passive system after we have applied the cycle $S_{m,n}$. In Supplementary Eq. (5) we have shown the probability distribution of $\tilde{\rho}_{\mathrm{P}}$ as a function of $\{q_i\}_{i=0}^{m+n-1}$. Thanks to the constraints introduced in Supplementary Eqs. (7) to (12), we can simplify the form of $\tilde{\rho}_{\mathrm{P}}$, so that we obtain

$$p_0' = p_0 + m\,\Delta P, \tag{38}$$
$$p_1' = p_1 - (m+n)\,\Delta P, \tag{39}$$
$$p_2' = p_2 + n\,\Delta P. \tag{40}$$

We can easily notice that the cycle acts on the passive state by modifying the original probabilities by multiples of

$$\Delta P = \frac{p_1\, q_{m+n-1}}{\mathrm{T}_1(m-1) + \frac{p_1}{p_2}\mathrm{T}_2(n-1)} \left(\left(\frac{p_1}{p_2}\right)^n - \left(\frac{p_0}{p_1}\right)^m \right). \tag{41}$$

The expression of the final state $\tilde{\rho}_{\mathrm{P}}$ allows us to understand how the cycle operates over the system when work is extracted. In particular, we can consider the evolution of the system in two different situations, linked to the two possible scenarios of Supplementary Eq. (37).

Suppose that H_{P} is such that $m\,\Delta E_{10} > n\,\Delta E_{21}$. Then, from the conditions in $R^+_{m,n}$, we can verify that $\Delta P > 0$, so that the map is depleting the population of the state $|1\rangle_{\mathrm{P}}$, while increasing the populations of both $|0\rangle_{\mathrm{P}}$ and $|2\rangle_{\mathrm{P}}$ (see Supplementary Figure 1). Work is extracted from the cycle since the energy gained while moving $m\,\Delta P$ from p_1 to p_0 is bigger than the energy paid to move $n\,\Delta P$ from p_1 to p_2. In Supplementary Note 5, we show that the entropy of the system has to increase during the transformation. This is achieved since p_1 gets closer to p_2 after the cycle.

Let us consider the case in which H_{P} is such that $m\,\Delta E_{10} < n\,\Delta E_{21}$. Then, from the conditions in $R^+_{m,n}$, we can verify that $\Delta P < 0$, so that the map is depleting the populations of the states $|0\rangle_{\mathrm{P}}$ and $|2\rangle_{\mathrm{P}}$, while increasing the populations of $|1\rangle_{\mathrm{P}}$ (see Supplementary Figure 1). Work is extracted from the cycle since the energy gained while moving $n\,\Delta P$ from p_2 to p_1 is bigger than the energy paid to move $m\,\Delta P$ from p_0 to p_1. Moreover, the entropy of the system increases since p_0 gets closer to p_1 after the cycle.

It is worth noting that the final state $\tilde{\rho}_{\mathrm{P}}$ can be active. This happen, in the case of $m\,\Delta E_{10} > n\,\Delta E_{21}$, when $p_1' < p_2'$. In the other case, we obtain a final active state if $p_0' < p_1'$. In these situations, not only are we able to extract work from the passive state ρ_{P} during the cycle, but we can also perform a local unitary operation (permuting $|1\rangle_{\mathrm{P}}$ and $|2\rangle_{\mathrm{P}}$ in the first case, and $|0\rangle_{\mathrm{P}}$ and $|1\rangle_{\mathrm{P}}$ in the second) which allows for additional work extraction. It is also possible for the final state of the system to be passive, and to still lie inside the activable region $R^+_{m,n}$. Due to the correlation created between system and machine, however, this state cannot be used again, at least not with the same machine.

Supplementary Note 2. WORK EXTRACTION FROM A GENERIC QUDIT PASSIVE STATE

Work extraction from a generic qudit passive state $\rho_{\mathrm{P}}^{(d)}$ (for any Hamiltonian $H_{\mathrm{P}}^{(d)}$) can be achieved with the cycle introduced in Supplementary Note 1, even if this work extraction is not optimal (as it might be when we deal with qutrit state, as we see in Supplementary Note 4). Indeed, even if the system has d levels, we only need to focus our analysis on three of them, and perform the cycle on these levels only. Thus, given the state $\rho_{\mathrm{P}}^{(d)} = \sum_{i=0}^{d-1} p_i \,|i\rangle\,\langle i|_{\mathrm{P}}$ and the Hamiltonian $H_{\mathrm{P}}^{(d)} = \sum_{i=0}^{d-1} E_i \,|i\rangle\,\langle i|_{\mathrm{P}}$, we can consider the subspace $\mathrm{A}_k = \mathrm{span}\left\{|k\rangle_{\mathrm{P}}, |k+1\rangle_{\mathrm{P}}, |k+2\rangle_{\mathrm{P}}\right\}$, for a given $k \in [0, d-3]$. Thus, we can divide the qudit state and the Hamiltonian in two contributions, one with support over A_k, the other with support over its complement,

$$\rho_{\mathrm{P}}^{(d)} = \left(\sum_{i\in \mathrm{A}_k} p_i\right) \rho_{\mathrm{P}}^{(\mathrm{A})} + \left(1 - \sum_{i\in \mathrm{A}_k} p_i\right) \rho_{\mathrm{P}}^{(\mathrm{A}^c)}, \tag{42}$$
$$H_{\mathrm{P}}^{(d)} = H_{\mathrm{P}}^{(\mathrm{A})} + H_{\mathrm{P}}^{(\mathrm{A}^c)}, \tag{43}$$

where the normalised quantum states are

$$\rho_{\mathrm{P}}^{(\mathrm{A})} = \sum_{i\in \mathrm{A}_k} \frac{p_i}{\sum_{j\in\mathrm{A}_k} p_j} \left|i\right\rangle \left\langle i\right|_{\mathrm{P}} , \tag{44}$$

$$\rho_{\mathrm{P}}^{(\mathrm{A}^c)} = \sum_{i\notin \mathrm{A}_k} \frac{p_i}{1-\sum_{j\in\mathrm{A}_k} p_j} \left|i\right\rangle \left\langle i\right|_{\mathrm{P}} , \tag{45}$$

while the Hamiltonian contributions are, respectively, $H_{\mathrm{P}}^{(\mathrm{A})} = \sum_{i\in\mathrm{A}_k} E_i \left|i\right\rangle\left\langle i\right|_{\mathrm{P}}$ and $H_{\mathrm{P}}^{(\mathrm{A}^c)} = \sum_{i\notin\mathrm{A}_k} E_i \left|i\right\rangle\left\langle i\right|_{\mathrm{P}}$. In the following, we define $\lambda = \sum_{i\in\mathrm{A}_k} p_i$, so that $\rho_{\mathrm{P}}^{(d)} = \lambda\, \rho_{\mathrm{P}}^{(\mathrm{A})} + (1-\lambda)\, \rho_{\mathrm{P}}^{(\mathrm{A}^c)}$.

We can now introduce an ancillary system (the machine M) of dimension $m+n$, described by the state ρ_{M}, together with the global unitary operator U,

$$U = P_{\mathrm{A}^c} \otimes \mathbb{I}_{\mathrm{M}} + P_{\mathrm{A}} \otimes \mathbb{I}_{\mathrm{M}} \circ S_{m,n} \circ P_{\mathrm{A}} \otimes \mathbb{I}_{\mathrm{M}}, \tag{46}$$

where the operator $S_{m,n}$, described in Supplementary Eq. (4), has support on $\mathrm{A}_k \otimes \mathcal{H}_{\mathrm{M}}$, and therefore commute with $P_{\mathrm{A}} \otimes \mathbb{I}_{\mathrm{M}}$. If we consider the evolution of the system under this operator, we obtain

$$\tilde{\rho}_{\mathrm{P}}^{(d)} = \mathrm{Tr}_{\mathrm{M}} \left[U \left(\rho_{\mathrm{P}}^{(d)} \otimes \rho_{\mathrm{M}} \right) U^\dagger \right] = \lambda\, \mathrm{Tr}_{\mathrm{M}} \left[S_{m,n} \left(\rho_{\mathrm{P}}^{(\mathrm{A})} \otimes \rho_{\mathrm{M}} \right) S_{m,n}^\dagger \right] + (1-\lambda)\, \rho_{\mathrm{P}}^{(\mathrm{A}^c)} = \lambda\, \tilde{\rho}_{\mathrm{P}}^{(\mathrm{A})} + (1-\lambda)\, \rho_{\mathrm{P}}^{(\mathrm{A}^c)}, \tag{47}$$

and we can easily verify, due to the properties of $S_{m,n}$, that the local state of the machine is left unchanged. The amount of work extracted during this cycle is

$$\Delta W = \mathrm{Tr}_{\mathrm{P}} \left[H_{\mathrm{P}}^{(d)} \left(\rho_{\mathrm{P}}^{(d)} - \tilde{\rho}_{\mathrm{P}}^{(d)} \right) \right] = \lambda\, \mathrm{Tr}_{\mathrm{P}} \left[H_{\mathrm{P}}^{(\mathrm{A})} \left(\rho_{\mathrm{P}}^{(\mathrm{A})} - \tilde{\rho}_{\mathrm{P}}^{(\mathrm{A})} \right) \right], \tag{48}$$

and the problem reduces to the one analysed at the beginning of this section (that is, to the extraction of work from a qutrit system described by the passive state $\rho_{\mathrm{P}}^{(\mathrm{A})}$, with Hamiltonian $H_{\mathrm{P}}^{(\mathrm{A})}$), with the only difference of a multiplicative factor $\lambda \in (0,1)$ in ΔW.

Supplementary Note 3. WORK EXTRACTION AND k-ACTIVABLE STATES

The set of passive states can be divided into a hierarchy of classes, which divides the states according to the number of copies needed to activate them. Here, we say that a state is active if it is not passive, and therefore if we can extract work from it with unitary operations. Any passive but not completely passive state can be activated if we tensor together enough copies of it. In particular, when k copies of a passive state are active, we call the state k-activable. We now show that, if work is extracted from a qutrit passive state ρ_{P}, with Hamiltonian H_{P}, through the cycle $S_{m,n}$, then the state realised by $m+n$ copies of ρ_{P} is active. It worth noting that, while our cycle only requires an additional system of dimension $m+n$ to extract work from ρ_{P}, in order to activate the same state we would need $m+n-1$ copies of it, that is, an ancilla whose size is exponential in $n+m$.

In the following, we consider a qutrit system, although the same argument applies to qudit systems, for the reasons presented in the previous section. If the passive state ρ_{P} is activated by the cycle $S_{m,n}$, then one of the two conditions in Supplementary Eq. (37) has to be satisfied. Let us assume that the conditions satisfied by state and Hamiltonian are

$$m\, \Delta E_{10} > n\, \Delta E_{21}, \tag{49}$$

$$\left(\frac{p_1}{p_2} \right)^n > \left(\frac{p_0}{p_1} \right)^m, \tag{50}$$

where the other case follows straightforwardly.

Consider now a system composed by $n+m$ copies of the qutrit system under examination, with Hamiltonian $H_{\mathrm{tot}} = \sum_{i=1}^{m+n} H_{\mathrm{P}}^{(i)}$, where the term $H_{\mathrm{P}}^{(i)}$ acts over the i-th copy. The state of this global system is $\rho_{\mathrm{P}}^{\otimes m+n}$. Then, let us focus our attention on two eigenstates of H_{tot}, namely, $\left|1\right\rangle^{\otimes m+n}$ and $\left|0\right\rangle^{\otimes m} \otimes \left|2\right\rangle^{\otimes n}$. The first eigenstate has an energy of $(m+n)\, E_1$, and its occupation probability is p_1^{m+n}. The second eigenstate, instead, has energy $m\, E_0 + n\, E_2$, and its occupation probability is $p_0^m p_2^n$. It is easy to verify that, if the constraints of Supplementary Eqs. (49) and (50) hold, then the inequalities $(m+n)\, E_1 > m\, E_0 + n\, E_2$ and $p_1^{m+n} > p_0^m p_2^n$ are satisfied, implying that the state $\rho_{\mathrm{P}}^{\otimes m+n}$ is active. Thus, we have shown that if a passive state ρ_{P} can be activated with the cycle $S_{m,n}$, then the state $\rho_{\mathrm{P}}^{\otimes m+n}$ is active. However, this result does not tell us whether it is possible to activate the state ρ_{P} by tensoring it with less copies. In the same way, we do not know whether the fact that the state $\rho_{\mathrm{P}}^{\otimes m+n}$ is active implies that we can extract work from ρ_{P} with the cycle $S_{m,n}$.

Supplementary Note 4. ASYMPTOTIC BEHAVIOUR OF THE MACHINE

We are now interested in the study of the cycle $S_{m,n}$ when the size of the machine (as well as the number of hot and cold swaps) tends to infinity. In particular, we are interested in the form of the probability distribution of the machine, the work extracted, and the final state of the passive system. Let us consider the Hamiltonian H_{P} of the main (qutrit) system. We know that, for any Hamiltonian H_{P}, there exists two integer numbers N and M such that $M\,\Delta E_{10} = N\,\Delta E_{21}$. We now consider a passive state ρ_{P} describing this system whose probability distribution satisfies $(p_1/p_2)^N > (p_0/p_1)^M$. Notice that this condition implies that the state is in the subset of passive states denoted by R_1 (see the main text, Fig. 5), or equivalently it implies that the hot virtual temperature is associated with the pair of states $|0\rangle_{\mathrm{P}}$ and $|1\rangle_{\mathrm{P}}$. One could analyse the opposite situation as well, but the results we obtain would be analogous, due to the symmetry of the problem with respect to the hot and cold interactions.

In order to perform the asymptotic expansion of the probability distribution of the machine, Supplementary Eqs. (32), (33), and (34), we first want to define how the ratio $\frac{n}{m}$ behaves as the number of hot and cold swaps goes to infinity. We set this fraction equal to α, so that $n = \alpha\, m$, and we define a range for this parameter, due to the constraints we set on the passive state. Indeed, if we want to extract work, we need m and n to satisfy one of the two conditions in Supplementary Eq. (37), and in particular, since we assume the passive state to be in the region R_1, we need $m\,\Delta E_{10} > n\,\Delta E_{21}$ and $(p_1/p_2)^n > (p_0/p_1)^m$. The two inequalities implies that

$$\frac{\log \frac{p_0}{p_1}}{\log \frac{p_1}{p_2}} < \alpha < \frac{\Delta E_{10}}{\Delta E_{21}}, \tag{51}$$

where it is easy to verify that the lower bound is smaller than the upper one, due to the fact that $\rho_{\mathrm{P}} \in R_1$.

We can now use the assumptions made on the cycle (that is, on the parameters m and n) and on the initial passive state in order to expand the probability distribution of ρ_{M} for $m, n \to \infty$. As a first step, let us consider the coefficient $D(m,n)$ presented in Supplementary Eq. (31). When m and n tends to infinity, we find that

$$D(m,n) \approx 1 + \left(\frac{p_0}{p_1}\right)^m \left(\frac{p_2}{p_1}\right)^n \frac{(p_0-p_2)\,(p_1-p_2)}{(p_0-p_1)} + O\left(\left(\frac{p_2}{p_1}\right)^n ; \left(\frac{p_0}{p_1}\right)^{2m} \left(\frac{p_2}{p_1}\right)^{2n}\right), \tag{52}$$

where it is easy to verify that the term $(p_0/p_1)^m\,(p_2/p_1)^n \to 0$ as $m, n \to \infty$, and that both $(p_2/p_1)^n$ and $(p_0/p_1)^{2m}\,(p_2/p_1)^{2n}$ tends to 0 faster that this first term. However, we cannot say which one is the fastest without further assumptions, and that is the reason we keep both in the O.

Once the expansion of $D(m,n)$ is known, we can focus on the probability distribution of the machine. For simplicity, we consider the distribution in Supplementary Eqs. (32), (33), and (34), where q_{m+n-1} is not defined yet; we will define it through the normalisation condition once the asymptotic expansion has been performed. We find that

$$q_j \approx q_{m+n-1} \left(\frac{p_0-p_2}{p_0-p_1} + O\left(\left(\frac{p_0}{p_1}\right)^m \left(\frac{p_2}{p_1}\right)^n\right)\right) \left(\frac{p_0}{p_1}\right)^{m-j} \quad ; \quad j = 0, \ldots, m-1, \tag{53}$$

$$q_j \approx q_{m+n-1} \left(\frac{p_2}{p_1}\frac{p_0-p_2}{p_0-p_1} + O\left(\left(\frac{p_0}{p_1}\right)^m \left(\frac{p_2}{p_1}\right)^n\right)\right) \left(\frac{p_0}{p_1}\right)^m \left(\frac{p_1}{p_2}\right)^{m-j} \quad ; \quad j = m, \ldots, m+n-3, \tag{54}$$

$$q_{m+n-2} \approx q_{m+n-1} \left(1 + O\left(\left(\frac{p_0}{p_1}\right)^m \left(\frac{p_2}{p_1}\right)^n\right)\right). \tag{55}$$

We are now able to obtain the value of q_{m+n-1} by imposing the normalisation condition over the asymptotic probability distribution of the machine. We find that

$$q_{m+n-1} \approx \left(\frac{(p_1-p_2)\,(p_0-p_1)^2}{p_1\,(p_0-p_2)^2} + O\left(\left(\frac{p_0}{p_1}\right)^m \left(\frac{p_2}{p_1}\right)^n\right)\right) \left(\frac{p_1}{p_0}\right)^m, \tag{56}$$

that is, q_{m+n-1} tends to 0 as $(p_1/p_0)^m$ for $m \to \infty$. Notice that the same result can be obtained by expanding Supplementary Eq. (35). If we send m and n to infinity, we find that the asymptotic probability distribution of the

machine is

$$q_j \approx \frac{(p_1 - p_2)(p_0 - p_1)}{p_1 (p_0 - p_2)} \left(\frac{p_0}{p_1}\right)^{-j} \quad ; \quad j = 0, \ldots, m-1, \tag{57}$$

$$q_j \approx \frac{p_2 (p_1 - p_2)(p_0 - p_1)}{p_1^2 (p_0 - p_2)} \left(\frac{p_1}{p_2}\right)^{m-j} \quad ; \quad j = m, \ldots, m+n-3, \tag{58}$$

$$q_{m+n-2} \approx q_{m+n-1} \approx \frac{(p_1 - p_2)(p_0 - p_1)^2}{p_1 (p_0 - p_2)^2} \left(\frac{p_1}{p_0}\right)^{m}. \tag{59}$$

We can now investigate how the probability distribution of the main system changes, and evaluate the asymptotic work extracted ΔW during on cycle. Let us consider the probability unit ΔP, introduced in Supplementary Eq. (41). If we set m and n to infinity, we have that

$$\Delta P \approx \frac{(p_1 - p_2)^2 (p_0 - p_1)^2}{p_1 (p_0 - p_2)^2} \left(\frac{p_1}{p_0}\right)^{m}, \tag{60}$$

that tends to 0 with an exponential scaling. Therefore, the heat engine with infinite-dimensional thermal machine only modifies the passive states by an infinitesimal amount. As a consequence, the work extracted has to be infinitesimal as well. Indeed, by considering Supplementary Eq. (36) it is easy to show that ΔW tends to 0 as $m, n \to \infty$, since ΔW is proportional to ΔP (modulo a multiplying factor proportional to m, which tends to infinity more slowly than $(p_1/p_0)^m$ tends to 0).

Final state and work extraction over multiple cycles

In the previous section we have seen that, when the machine is infinitely large, we only modify the passive state infinitesimally. We can then consider the situation in which we are given an infinite number of these machines, and we want to evolve the passive state (and extract work) by sequentially applying our cycle with the help of these machines. In order to study the evolution of the passive state, we can consider its probability distribution after one cycle, see Supplementary Eqs. (38), (39), and (40). These equations can be recast as differential equations, since $\Delta P \to 0$ in this scenario. It is easy to verify that the differential equations which govern the evolution of the passive state are

$$\frac{\mathrm{d}p_0}{\mathrm{d}t} = \frac{(p_1 - p_2)^2 (p_0 - p_1)^2}{p_1 (p_0 - p_2)^2}, \tag{61}$$

$$\frac{\mathrm{d}p_1}{\mathrm{d}t} = -(1+\alpha) \frac{(p_1 - p_2)^2 (p_0 - p_1)^2}{p_1 (p_0 - p_2)^2}, \tag{62}$$

where $\alpha = \frac{n}{m}$ takes values in the range given by Supplementary Eq. (51), and we define

$$\frac{\mathrm{d}p_i}{\mathrm{d}t} = \lim_{m \to \infty} \frac{p_i' - p_i}{\Delta p(m)} \quad , \quad \Delta p(m) = m \left(\frac{p_1}{p_0}\right)^{m} \quad \text{for } i = 0, 1, \tag{63}$$

with $\{p_0, p_1, 1 - p_0 - p_1\}$ the probability distribution of the state before the cycle, and $\{p_0', p_1', 1 - p_0' - p_1'\}$ the distribution of the state after the cycle. The continuous parameter t is here related to the number of cycles we perform on the system. It is worth noting that Supplementary Eqs. (61) and (62) share a common (positive) factor. Therefore we have that, as time goes on, the probability of occupation of $|0\rangle_{\mathrm{P}}$ increases, while the one of $|1\rangle_{\mathrm{P}}$ decreases (as expected from the discussion in Supplementary Note 1). Moreover, since $\alpha > 0$, the increase in the former is slower than the decreasing of the latter.

The two differential equations can be reshaped in a single, more helpful one,

$$\frac{\mathrm{d}p_1}{\mathrm{d}t} = -(1+\alpha) \frac{\mathrm{d}p_0}{\mathrm{d}t}, \tag{64}$$

and we can investigate the solution of this equation for α close to its limiting values. As a first step, let us consider the case in which $\alpha = \frac{\Delta E_{10}}{\Delta E_{12}} - \frac{1}{m} \approx \frac{\Delta E_{10}}{\Delta E_{12}}$. Then, the solution of Supplementary Eq. (64) is

$$p_1(t) = -\left(1 + \frac{\Delta E_{10}}{\Delta E_{12}}\right) \big(p_0(t) - p_0(t=0)\big) + p_1(t=0), \tag{65}$$

where $\{p_0(t), p_1(t), 1-p_0(t)-p_1(t)\}$ is the probability distribution of the state of the system at time t, and $t=0$ is the initial time (when the system is in $\rho_{\rm P}$). If we rearrange Supplementary Eq. (65), we see that it is equivalent to the following constraint for the evolved state

$$\mathrm{Tr}\left[H_{\rm P}\,\rho_{\rm P}(t)\right] = \mathrm{Tr}\left[H_{\rm P}\,\rho_{\rm P}\right] \quad \forall\, t \geq 0, \tag{66}$$

that is, the evolution conserves the energy of the system (equivalently, no work is extracted during the evolution). It is easy to see, for instance by representing the solution of Supplementary Eq. (65) in a two-dimensional plot of p_1 versus p_0, that the passive state is moving toward the set of thermal states, that are the steady states of this evolution. In fact, when a thermal state is considered, we find that $(p_1/p_2)^{\alpha} = (p_0/p_1)$, which implies $\Delta P = 0$. Thus, after enough time t is passed, we find that the initial passive state $\rho_{\rm P}$ has been mapped into the thermal state with inverse temperature $\beta_{\rm min}$, where

$$\beta_{\rm min} \;:\; \mathrm{Tr}\left[H_{\rm P}\,\tau_{\beta_{\rm min}}\right] = \mathrm{Tr}\left[H_{\rm P}\,\rho_{\rm P}\right] \quad , \quad \tau_{\beta_{\rm min}} = \frac{e^{-\beta_{\rm min} H_{\rm P}}}{Z_{\rm min}}, \tag{67}$$

and $Z_{\rm min}$ is the partition function of the system at temperature $\beta_{\rm min}^{-1}$.

We can now consider the case in which $\alpha = \frac{\log p_0 - \log p_1}{\log p_1 - \log p_2} + \frac{1}{m} \approx \frac{\log p_0 - \log p_1}{\log p_1 - \log p_2}$, that is, when its value is close to its lower bound. We notice that, in this case, α itself depends on the probability distribution of the passive state. Then, if we replace α with its lower bound in Supplementary Eq. (64) we obtain

$$\log p_0 \,\frac{\mathrm{d}p_0}{\mathrm{d}t} + \log p_1 \,\frac{\mathrm{d}p_1}{\mathrm{d}t} + \log p_2 \,\frac{\mathrm{d}p_2}{\mathrm{d}t} = 0, \tag{68}$$

which, if integrated between time 0 and time t, gives the following constraint on the entropy of the evolved states

$$S\left(\rho_{\rm P}(t)\right) = S\left(\rho_{\rm P}\right) \quad \forall\, t \geq 0, \tag{69}$$

where $S(\rho) = -\mathrm{Tr}\left[\rho \log \rho\right]$ is the Von Neumann entropy. Therefore, the evolution of the passive state has to preserve the entropy of the system, and the state is moving toward the set of thermal states. For $t \to \infty$, the system is in the thermal state with inverse temperature $\beta_{\rm max}$, where

$$\beta_{\rm max} \;:\; S\left(\tau_{\beta_{\rm max}}\right) = S\left(\rho_{\rm P}\right) \quad , \quad \tau_{\beta_{\rm max}} = \frac{e^{-\beta_{\rm max} H_{\rm P}}}{Z_{\rm max}}, \tag{70}$$

and $Z_{\rm max}$ is the partition function of the system at temperature $\beta_{\rm max}^{-1}$.

Thus, when we set α equal to its limiting values, the evolution of the passive state can either follow a trajectory in which energy is conserved, or in which entropy is conserved. However, all intermediate trajectories can be achieved by imposing a different α inside the range specified by Supplementary Eq. (51), and consequently all passive states with lower or equal energy, and greater or equal entropy that $\rho_{\rm P}$ can be reached.

Supplementary Note 5. ACTIVATION MAPS

Consider a specific family of CPT maps which allow for work extraction from a system described by a passive state. The maps of this family, which we call activation maps, can be represented by unitary operations acting globally on both the main system and an ancilla, such that the local state of the ancillary system is preserved. The cycle of Supplementary Note 1 is a particular instance of these activation maps, and in the following we study the main properties of this family. Let us consider a system S with Hamiltonian $H_{\rm S}$, described by the state $\rho_{\rm S}$ (this state does not need to be passive). The energy that we extract from the system when we evolve it with the unitary operator $U_{\rm S}$ is given by the difference in average energy between the initial and final state,

$$\Delta W_{\rm S} = \mathrm{Tr}_{\rm S}\left[H_{\rm S}\left(\rho_{\rm S} - U_{\rm S}\,\rho_{\rm S}\,U_{\rm S}^{\dagger}\right)\right]. \tag{71}$$

We assume this energy to be stored in an implicit battery, and we refer to it as work. If the state is passive, then $\Delta W_{\rm S} \leq 0$, that is, we cannot extract work. If the state is active, we can find some unitary operations that allow for a positive work extraction. In particular, the maximum work we can extract is

$$\Delta W_{\rm S}^{\rm max} = \mathrm{Tr}_{\rm S}\left[H_{\rm S}\left(\rho_{\rm S} - \rho_{\rm S}^{\rm pass}\right)\right], \tag{72}$$

where the state $\rho_{\mathrm{S}}^{\mathrm{pass}}$ is the passive state obtained from the initial state ρ_{S}. In the literature, $\Delta W_{\mathrm{S}}^{\mathrm{max}}$ is known as ergotropy, see Supplementary Ref. [1]. This quantity is 0 if the initial state is passive, and positive otherwise.

We now add an ancillary system A with a trivial Hamiltonian, described by the state σ_{A}, and we consider the family of maps

$$\Lambda\left(\rho_{\mathrm{S}}\right)=\mathrm{Tr}_{\mathrm{A}}\left[U_{\mathrm{SA}}\left(\rho_{\mathrm{S}}\otimes\sigma_{\mathrm{A}}\right)U_{\mathrm{SA}}^{\dagger}\right], \tag{73}$$

where the unitary operator U_{SA} acts globally over system and ancilla, and we require that the final local state of the ancilla is equal to the initial one, that is,

$$\sigma_{\mathrm{A}}=\mathrm{Tr}_{\mathrm{S}}\left[U_{\mathrm{SA}}\left(\rho_{\mathrm{S}}\otimes\sigma_{\mathrm{A}}\right)U_{\mathrm{SA}}^{\dagger}\right]. \tag{74}$$

Notice that the global evolution can create correlations between system and ancilla, and our sole constraint regards the local state of the ancilla. The work extracted during the evolution is given by

$$\Delta W_{\mathrm{SA}}=\mathrm{Tr}_{\mathrm{S}}\left[H_{S}\left(\rho_{\mathrm{S}}-\Lambda(\rho_{\mathrm{S}})\right)\right], \tag{75}$$

where the only contribution is given by the energy difference in the system, due to the absence of any interaction term between system and ancilla, and to the fact that the final state of the ancilla is equal to its initial one.

We can now introduce the notion of activation of a quantum state,

Definition 1. Let us consider a system S with Hamiltonian H_{S}, described by the state ρ_{S}. Then, we say that ρ_{S} can be activated iff there exists an ancillary system A with trivial Hamiltonian, described by the state σ_{A}, and an activation map Λ as in Supplementary Eq. (73), satisfying the condition of Supplementary Eq. (74), such that

$$\Delta W_{\mathrm{SA}}>\Delta W_{\mathrm{S}}^{\mathrm{max}} \tag{76}$$

that is, if we can extract more work from ρ_{S} by acting with Λ than we can do by acting with any unitary operation.

As we noticed before, an example of activation map is the one used in our passive engine, Supplementary Eq. (5), where the ancillary system is the machine, and the global unitary operation is $S_{m,n}$.

General properties of the final state of an activation map

Although the family of maps introduced in the previous section is extremely general, we can still use their definition to derive some properties of the final state $\Lambda\left(\rho_{\mathrm{S}}\right)$. The first, trivial property consists in the fact that the final state of an activation map has to have a lower energy than the one possessed by a the passified version of the initial state,

$$\mathrm{Tr}_{\mathrm{S}}\left[H_{\mathrm{S}}\,\rho_{\mathrm{S}}^{\mathrm{pass}}\right]>\mathrm{Tr}_{\mathrm{S}}\left[H_{\mathrm{S}}\,\Lambda\left(\rho_{\mathrm{S}}\right)\right], \tag{77}$$

where this condition is obtained by replacing Supplementary Eqs. (72) and (75) into Def. 1.

A second property regards the entropy of the final state. Due to the invariance of Von Neumann entropy under unitary operations, its sub-additivity, and the constraint on the local state of the machine, Supplementary Eq. (74), we can show that

$$S(\rho_{\mathrm{S}})\leq S(\Lambda\left(\rho_{\mathrm{S}}\right)), \tag{78}$$

that is, the entropy of the system cannot decrease during the evolution through Λ, and it increases if correlations create between system and machine.

If we use the two constraints on $\Lambda\left(\rho_{\mathrm{S}}\right)$ together, we can show that any completely passive state cannot be activated. In this case, in fact, we have that $\rho_{\mathrm{S}}=\rho_{\mathrm{S}}^{\mathrm{pass}}=\tau_{\beta}$, that is, the state under examination is the thermal state of Hamiltonian H_{S} for a certain $\beta\in[0,\infty]$. But we know that this state is the one with minimum energy for a given entropy, or, vice versa, the one with maximum entropy for given energy. Then, we cannot find another state $\Lambda\left(\rho_{\mathrm{S}}\right)$ such that the two conditions of Supplementary Eqs. (77) and (78) are satisfied at the same time. This implies that any completely passive state cannot be activated, pure ground state and maximally-mixed state included.

We can also consider a generic pure state $\rho_{\mathrm{S}}=\left|\psi\right\rangle\left\langle\psi\right|$. The corresponding passified state is the ground state $\left|0\right\rangle$. From Supplementary Eq. (77) it follows that the final state of Λ has to have a lower energy than $\rho_{\mathrm{S}}^{\mathrm{pass}}$. But since the passified state we obtain, $\left|0\right\rangle$, is by definition the state with minimum energy, we cannot satisfy this condition. Thus, we cannot activate, in the sense of Def. 1, any pure state $\left|\psi\right\rangle$.

Asymptotic work extraction from passive states

It was proved by Alicki *et al.* (Supplementary Ref. [2]) that, when an infinite number of copies of a passive state $\rho_{\rm S}$ are considered, the optimal extractable work per single copy is given by

$$\Delta W_{\rm opt} = {\rm Tr}_{\rm S}\left[H_{\rm S}\left(\rho_{\rm S} - \tau_{\beta_{\rm max}}\right)\right], \tag{79}$$

where $\tau_{\beta_{\rm max}}$ is the thermal state with inverse temperature $\beta_{\rm max}$ such that $S(\tau_{\beta_{\rm max}}) = S(\rho_{\rm S})$. We want to compare the work extracted in the asymptotic limit with the work extracted with a generic activation map Λ. This comparison can be easily carried out using the main properties of the final state $\Lambda(\rho_{\rm S})$, see Supplementary Eqs. (77) and (78), together with the properties of $\tau_{\beta_{\rm max}}$.

For any given final state of the system $\Lambda(\rho_{\rm S})$, there always exists an inverse temperature $\hat{\beta}$, and a thermal state $\tau_{\hat{\beta}}$ at that temperature, such that $S(\tau_{\hat{\beta}}) = S\left(\Lambda(\rho_{\rm S})\right)$. Since the state $\tau_{\hat{\beta}}$ is thermal, we have that its energy is minimum, that is,

$$ {\rm Tr}_{\rm S}\left[H_{\rm S}\,\Lambda(\rho_{\rm S})\right] \geq {\rm Tr}_{\rm S}\left[H_{\rm S}\tau_{\hat{\beta}}\right]. \tag{80}$$

Moreover, from Supplementary Eq. (78) it follows that the entropy of $\tau_{\hat{\beta}}$ is greater than the entropy of the state $\tau_{\beta_{\rm max}}$, introduced in the previous paragraph. By considering this entropic condition together with the free energy difference $F_{\beta_{\rm max}}(\tau_{\hat{\beta}}) - F_{\beta_{\rm max}}(\tau_{\beta_{\rm max}}) \geq 0$, we obtain that the state $\tau_{\hat{\beta}}$ is more energetic than $\tau_{\beta_{\rm max}}$, that is,

$$ {\rm Tr}_{\rm S}\left[H_{\rm S}\tau_{\hat{\beta}}\right] \geq {\rm Tr}_{\rm S}\left[H_{\rm S}\tau_{\beta_{\rm max}}\right]. \tag{81}$$

From the above inequalities we have that

$$\Delta W_{\rm opt} - \Delta W_{\rm SA} = {\rm Tr}_{\rm S}\left[H_{\rm S}\,\Lambda(\rho_{\rm S})\right] - {\rm Tr}_{\rm S}\left[H_{\rm S}\tau_{\beta_{\rm max}}\right] \geq {\rm Tr}_{\rm S}\left[H_{\rm S}\tau_{\hat{\beta}}\right] - {\rm Tr}_{\rm S}\left[H_{\rm S}\tau_{\beta_{\rm max}}\right] \geq 0, \tag{82}$$

where the first inequality follows from Supplementary Eq. (80), while the second follows from Supplementary Eq. (81). Therefore, the energy we extract with the aid of an activation map Λ is always equal or lower than the energy (per single copy) that we extract by acting over an infinite number of copies of the passive state with a global unitary operator, $\Delta W_{\rm opt} \geq \Delta W_{\rm SA}$.

Thus, $\Delta W_{\rm opt}$ is an upper bound for the work extracted by any activation map Λ. In Supplementary Refs. [2, 3] it was shown that this upper bound can be actually achieved by acting over infinite many copies of the system with a global unitary operation. In this paper, instead, we have shown that the extraction of an amount of work equal to $\Delta W_{\rm opt}$ is also achievable by acting on a single copy of the state. However, one needs to utilise infinite many infinite-dimensional machines to do so, as we showed in Supplementary Note 4.

Ancilla as part of a bigger thermal bath

Consider the case in which the ancilla utilised in Λ is just a subsystem of an infinite thermal reservoir at temperature β^{-1}. In this situation, we have to explicitly define an Hamiltonian $H_{\rm A}$ (where we have the freedom to rigidly translate the spectrum of this Hamiltonian), so that the state of the ancilla $\sigma_{\rm A}$ coincides with the thermal state $\tau_{\beta}^{({\rm A})} = e^{-\beta H_{\rm A}}/Z_{\rm A}$.

As we have seen, the map Λ lowers the energy of the system and builds correlations between system and ancilla, while preserving the local state of the ancillary system. If we consider the ancilla as part of the infinite bath, then we see that Λ extracts work from the passive state while no heat is exchanged with the bath (as the local state of the ancilla is unchanged). In the following we show that the energy extracted during this transformation is always lower than the difference in free energy between the initial state $\rho_{\rm S}$ and the thermal state $\tau_{\beta}^{({\rm S})} = e^{-\beta H_{\rm S}}/Z_{\rm S}$. Even in the case in which Λ maps $\rho_{\rm S}$ into $\tau_{\beta}^{({\rm S})}$, the work extracted is not optimal, as part of this work is locked inside the correlations between system and ancilla. In order to extract the remaining work from the correlations, and thus to perform optimal work extraction, we have to exploit the infinite thermal reservoir, exchanging an amount of heat proportional to the difference in entropy between $\tau_{\beta}^{({\rm S})}$ and $\rho_{\rm S}$. It is worth noting that, although this second operation allows us to extract an higher amount of work than the one obtained with the sole Λ, we do not consider it as an allowed operation in our framework, as it requires an additional ancillary system (the bath) with infinite dimension.

During the first operation we map the initial state $\rho_{\rm S}$ into the final one $\Lambda(\rho_{\rm S})$. This final state might or might not be a thermal state of $H_{\rm S}$, and the sole constraints we have are given by Supplementary Eqs. (77) and (78) (energy

has to decrease while entropy has to increase). The work we extract is the energy difference between the initial and final state, as we show in Supplementary Eq. (75),

$$\Delta W_1 = \mathrm{Tr}_{\mathrm{S}}\left[H_S\left(\rho_{\mathrm{S}} - \Lambda(\rho_{\mathrm{S}})\right)\right], \tag{83}$$

which is positive by definition, since we assume Λ to be an activation map, see Def. 1. The final state of system and ancilla is $\tilde{\rho}_{\mathrm{SA}} = U_{\mathrm{SA}}\left(\rho_{\mathrm{S}} \otimes \tau_\beta^{(\mathrm{A})}\right) U_{\mathrm{SA}}^\dagger$, and correlations are present, quantified by the mutual information

$$I\left(\tilde{\mathrm{S}} : \tilde{\mathrm{A}}\right) = S(\Lambda(\rho_{\mathrm{S}})) + S(\tau_\beta^{(\mathrm{A})}) - S(\tilde{\rho}_{\mathrm{SA}}). \tag{84}$$

The heat Q_1 exchanged during this transformation is equal to 0, as the local state of the bath does not change.

We now use the power of the infinite thermal reservoir to extract the last part of work from the state $\tilde{\rho}_{\mathrm{SA}}$, by mapping it into $\tau_\beta^{(\mathrm{S})} \otimes \tau_\beta^{(\mathrm{A})}$. In this case, work is given by the free energy difference between the two states, that is

$$\Delta W_2 = F_\beta\left(\tilde{\rho}_{\mathrm{SA}}\right) - F_\beta\left(\tau_\beta^{(\mathrm{S})} \otimes \tau_\beta^{(\mathrm{A})}\right) = \frac{1}{\beta}\left(D\left(\Lambda(\rho_{\mathrm{S}})||\tau_\beta^{(\mathrm{S})}\right) + I\left(\tilde{\mathrm{S}} : \tilde{\mathrm{A}}\right)\right), \tag{85}$$

where $D\left(\Lambda(\rho_{\mathrm{S}})||\tau_\beta^{(\mathrm{S})}\right) = \beta\left(F_\beta\left(\Lambda(\rho_{\mathrm{S}})\right) - F_\beta\left(\tau_\beta^{(\mathrm{S})}\right)\right)$ is the relative entropy between $\Lambda(\rho_{\mathrm{S}})$ and $\tau_\beta^{(\mathrm{S})}$. Since both the relative entropy and the mutual information are non-negative quantities, we have that work is indeed extracted during this second process. The heat exchanged in this second transformation is equal to the entropy difference (modulo the multiplicative constant β^{-1}) between the final and initial state

$$Q_2 = \frac{1}{\beta}\left(S\left(\tau_\beta^{(\mathrm{S})} \otimes \tau_\beta^{(\mathrm{A})}\right) - S\left(\tilde{\rho}_{\mathrm{SA}}\right)\right) = \frac{1}{\beta}\left(S\left(\tau_\beta^{(\mathrm{S})}\right) - S\left(\rho_S\right)\right), \tag{86}$$

where the last equality follows from the invariance under unitary operations of the Von Neumann entropy.

If we now consider the two transformations as a single one, we see that the total work extracted is

$$\Delta W_{\mathrm{tot}} = \Delta W_1 + \Delta W_2 = F_\beta\left(\rho_S\right) - F_\beta\left(\tau_\beta^{(\mathrm{S})}\right), \tag{87}$$

that is, ΔW_{tot} is optimal, and the heat exchanged is Q_2, equal to the entropy difference between $\tau_\beta^{(\mathrm{S})}$ and ρ_{S}.

An interesting scenario occurs when Λ maps the initial state into $\tau_\beta^{(\mathrm{S})}$. In this case, we see that the work we obtain in the second transformation (the one involving the whole thermal bath) is proportional to the sole mutual information, so that work is exclusively extracted from the correlations between system and catalyst. The amount of work in this case (see also Supplementary Ref. [4], Sec. VI B) is

$$\Delta W_2^{\mathrm{corr}} = \frac{1}{\beta} I\left(\tilde{\mathrm{S}} : \tilde{\mathrm{A}}\right) = \frac{1}{\beta}\left(S(\tau_\beta^{(\mathrm{S})}) - S(\rho_{\mathrm{S}})\right), \tag{88}$$

where the quantity is still non-negative, since Λ can map ρ_{S} into $\tau_\beta^{(\mathrm{S})}$ only if $S(\rho_{\mathrm{S}}) \leq S(\tau_\beta^{(\mathrm{S})})$, see Supplementary Note 5.

Supplementary Note 6. TECHNICAL RESULTS

In this section we show some of the technical results we have used to analyse the generic cycle on passive states.

Technical Result 2. Consider the sequence of real numbers $\{x_j\}_a^b$, those elements are linked by the following set of equations,

$$x_j = (1+\lambda)\, x_{j+1} - \lambda\, x_{j+2} \quad ; \quad j = a, \dots, b-2,$$

where $\lambda \in \mathbb{R}$ and $a, b \in \mathbb{N}$, $a \leq b-2$. Then, the elements of this sequence can be expressed in terms of x_{b-1} and x_b as

$$x_j = \mathrm{T}(b-(j+1), \lambda)\, x_{b-1} - \lambda\, \mathrm{T}(b-(j+2), \lambda)\, x_b \quad ; \quad j = a, \dots, b-2,$$

where $\mathrm{T}(h, \lambda) = \sum_{l=0}^{h} \lambda^l = \frac{1-\lambda^{h+1}}{1-\lambda}$.

Proof. If we insert the solution into the set of equations, we find

$$\mathrm{T}(b-(j+1),\lambda)\,x_{b-1}-\lambda\,\mathrm{T}(b-(j+2),\lambda)\,x_b=(1+\lambda)\mathrm{T}(b-(j+2),\lambda)\,x_{b-1}-\lambda(1+\lambda)\,\mathrm{T}(b-(j+3),\lambda)\,x_b\\ -\lambda\,\mathrm{T}(b-(j+3),\lambda)\,x_{b-1}+\lambda^2\,\mathrm{T}(b-(j+4),\lambda)\,x_b$$

for j taking values from a to $b-2$. We can re-organise the above equation, and we find that it is satisfied iff

$$\mathrm{T}(b-(j+1),\lambda)=(1+\lambda)\,\mathrm{T}(b-(j+2),\lambda)-\lambda\,\mathrm{T}(b-(j+3),\lambda)\quad;\quad j=a,\ldots,b-2, \tag{89}$$

$$\mathrm{T}(0,\lambda)=(1+\lambda)\,\mathrm{T}(-1,\lambda)-\lambda\,\mathrm{T}(-2,\lambda). \tag{90}$$

These two equalities easily follow from the definition of $\mathrm{T}(h,\lambda)$, as it can be check by replacing this coefficient with its explicit form in both Supplementary Eq. (89) and (90). □

Technical Result 3. The probability distribution of the state ρ_{M} is positive and normalised.

Proof. Let us consider the probabilities q_j for $j=0,\ldots,m-1$, as given in Supplementary Eq. (32). If we replace j with $j'=m-j$, then the main coefficient in the equation becomes

$$\mathrm{T}_1(j')-\frac{p_2}{p_1}\,D(m,n)\,\mathrm{T}_1(j'-1)=\frac{\mathrm{T}_1(j')\,\mathrm{T}_1(m-1)-\mathrm{T}_1(j'-1)\,\mathrm{T}_1(m)}{\mathrm{T}_1(m-1)+\frac{p_1}{p_2}\mathrm{T}_2(n-1)}\\ +\frac{p_1}{p_2}\,\frac{\mathrm{T}_1(j')\,\mathrm{T}_2(n-1)-\mathrm{T}_1(j'-1)\,\mathrm{T}_2(n-2)}{\mathrm{T}_1(m-1)+\frac{p_1}{p_2}\mathrm{T}_2(n-1)}.$$

It is clear that the denominator is positive, as $\mathrm{T}_1(h)$ and $\mathrm{T}_2(h)$ are positive for all $h\in\mathbb{Z}$. We need to show that the nominator is positive as well. The nominator of the first term can be reduced to

$$\mathrm{T}_1(j')\,\mathrm{T}_1(m-1)-\mathrm{T}_1(j'-1)\,\mathrm{T}_1(m)=\mathrm{T}_1(m-1)-\mathrm{T}_1(j'-1)=\sum_{l=j'}^{m-1}\left(\frac{p_0}{p_1}\right)^l\geq 0,$$

where the last equality follows from the fact that $j'=1,\ldots,m$. The nominator of the second term can be expressed as

$$\mathrm{T}_1(j')\,\mathrm{T}_2(n-1)-\mathrm{T}_1(j'-1)\,\mathrm{T}_2(n-2)=\mathrm{T}_1(j'-1)\left(\frac{p_1}{p_2}\right)^{n-1}+\mathrm{T}_2(n-2)\left(\frac{p_0}{p_1}\right)^{j'}+\left(\frac{p_0}{p_1}\right)^{j'}\left(\frac{p_1}{p_2}\right)^{n-1}>0.$$

Thus, the probabilities $\{q_j\}_{j=0}^{m-1}$ are positive when q_{m+n-1} is positive.

We can now focus on the probabilities q_j for $j=m,\ldots,m+n-3$, as given in Supplementary Eq. (33). By replacing j with $j'=m+n-(j+2)$ we obtain that the main coefficient in the equation becomes

$$\mathrm{T}_2(j')\,D(m,n)-\frac{p_1}{p_2}\,\mathrm{T}_2(j'-1)=\left(\frac{p_1}{p_2}\right)\frac{\mathrm{T}_2(j')\,\mathrm{T}_1(m)-\mathrm{T}_2(j'-1)\,\mathrm{T}_1(m-1)}{\mathrm{T}_1(m-1)+\frac{p_1}{p_2}\mathrm{T}_2(n-1)}\\ +\left(\frac{p_1}{p_2}\right)^2\frac{\mathrm{T}_2(j')\,\mathrm{T}_2(n-2)-\mathrm{T}_2(j'-1)\,\mathrm{T}_2(n-1)}{\mathrm{T}_1(m-1)+\frac{p_1}{p_2}\mathrm{T}_2(n-1)}.$$

As before, the denominator is positive, as $\mathrm{T}_1(h)$ and $\mathrm{T}_2(h)$ are both positive $\forall\, h\in\mathbb{Z}$. The nominator of the first term can be reduced to

$$\mathrm{T}_2(j')\,\mathrm{T}_1(m)-\mathrm{T}_2(j'-1)\,\mathrm{T}_1(m-1)=\mathrm{T}_2(j'-1)\left(\frac{p_0}{p_1}\right)^m+\mathrm{T}_1(m-1)\left(\frac{p_1}{p_2}\right)^{j'}+\left(\frac{p_1}{p_2}\right)^{j'}\left(\frac{p_0}{p_1}\right)^m>0.$$

The nominator of the second term can be expressed as

$$\mathrm{T}_2(j')\,\mathrm{T}_2(n-2)-\mathrm{T}_2(j'-1)\,\mathrm{T}_2(n-1)=\mathrm{T}_2(n-2)-\mathrm{T}_2(j'-1)=\sum_{l=j'}^{n-2}\left(\frac{p_1}{p_2}\right)^l\geq 0,$$

where the last equality follows from the fact that $j'=1,\ldots,n-2$. Thus, the probabilities $\{q_j\}_{j=m}^{m+n-3}$ are positive when $q_{m+n-1}>0$.

In Supplementary Eq. (34), we showed that q_{m+n-2} is related to q_{m+n-1} by the multiplicative coefficient $D(m,n)$, which can be easily shown to be positive for any integer $m,n\geq 1$. Finally, the normalisation condition force $q_{m+n-1}>0$, and implies the probability distribution of ρ_{M} to be positive and normalised. □

SUPPLEMENTARY REFERENCES

[1] Allahverdyan, A. E. , Balian, R. & Nieuwenhuizen, T. M. Maximal work extraction from finite quantum systems. *Europhysics Letters*, **67**, 565–571 (2004).
[2] Alicki, R. & Fannes, M. Entanglement boost for extractable work from ensembles of quantum batteries. *Physical Review E*, **87**, 042123 (2013).
[3] Sparaciari, C., Oppenheim, J. & Fritz, T. A resource theory for work and heat. Preprint at https://arxiv.org/abs/1607.01302 (2016).
[4] Perarnau-Llobet, M., et al. Extractable work from correlations. *Physical Review X*, **5**, 041011 (2015).
